# Exopolysaccharides from Marine and Marine Extremophilic Bacteria: Structures, Properties, Ecological Roles and Applications

**DOI:** 10.3390/md16020069

**Published:** 2018-02-20

**Authors:** Angela Casillo, Rosa Lanzetta, Michelangelo Parrilli, Maria Michela Corsaro

**Affiliations:** Department of Chemical Sciences, University of Naples “Federico II”, 80126 Naples, Italy; angela.casillo@unina.it (A.C.); lanzetta@unina.it (R.L.); parrilli@unina.it (M.P.)

**Keywords:** exopolysaccharides, chemical characterization, capsular polysaccharide, purification, EPS, extremophile, marine, NMR, GC-MS, structure/activity relationship

## Abstract

The marine environment is the largest aquatic ecosystem on Earth and it harbours microorganisms responsible for more than 50% of total biomass of prokaryotes in the world. All these microorganisms produce extracellular polymers that constitute a substantial part of the dissolved organic carbon, often in the form of exopolysaccharides (EPS). In addition, the production of these polymers is often correlated to the establishment of the biofilm growth mode, during which they are important matrix components. Their functions include adhesion and colonization of surfaces, protection of the bacterial cells and support for biochemical interactions between the bacteria and the surrounding environment. The aim of this review is to present a summary of the status of the research about the structures of exopolysaccharides from marine bacteria, including capsular, medium released and biofilm embedded polysaccharides. Moreover, ecological roles of these polymers, especially for those isolated from extreme ecological niches (deep-sea hydrothermal vents, polar regions, hypersaline ponds, etc.), are reported. Finally, relationships between the structure and the function of the exopolysaccharides are discussed.

## 1. Introduction

Polysaccharides are the most abundant organic material in the world. They are ubiquitous, as they can be recovered from plants, animals, algae and microorganisms in soil, water and atmosphere. The chemical structure of a polysaccharide comprises monomers called monosaccharides (Table 1), linked to each other through glycosidic linkages. They can be constituted of one type of monosaccharide, in homopolysaccharides, or by several types, usually up to ten, in heteropolysaccharides. They can display either a regular repeating unit, or random, or block distribution of monosaccharides. Various types of inorganic and organic substituents (sulphates, phosphates, acetates, ethers, amino acids, lactates and pyruvates) can decorate the polysaccharide backbone that in turn can be linear or branched. Arms can show different lengths and include the same monosaccharide types or different ones. Additionally, branches can be distributed in a regular or random way on the backbone. Finally, polysaccharides sizes range between 50 Da up to several thousand KDa.

With this high grade of variableness about the primary structure, it is easy to expect a huge number of polysaccharide structures that can be find in nature. These structures determine particular properties, with a precise biological function for the microorganism life that we are now beginning to understand [1].

Polysaccharides have industrial applications in the paper, food, biotechnological, textile, cosmetic and pharmaceutical industry due to their properties, such as mechanical strength, emulsifying, adhesive, rheological, metal-complexing, hydrocolloids forming. Nowadays, polysaccharides represent a possible ecological substitute of petroleum industry, as their sources are renewable and not pollutant. In addition, they are biodegradable, usually not toxic and their enzymatic or chemical degradation can produce biofuel together with many simple molecules, which are able to form industrial polymers and fine chemicals [2].

The common sources of industrial polysaccharide are plants, animals, fungi, algae and bacteria. However, fungi and bacteria have the advantage to give a high structure reproducibility due to the severely monitored conditions of microorganism growth. This is not possible for plant and animal sources, for which the polysaccharide structures depend on climate, environmental and feed conditions. According to these preliminary remarks, the research of new microorganisms for obtaining polysaccharides, with novel structures and properties virtually useful for new or improved industrial applications, is nowadays demanding.

Microorganisms populate every niche on Earth. In particular, marine habitats represent a large and unique environment where diverse communities of bacteria perform fundamental activities for the survival of planet’s ecosystem [3]. Oceans microorganisms account for approximately half of the primary production of organic substances on earth [4] and, among these, exopolysaccharides occupy a prominent position. These polymers are included into the dissolved organic carbon (DOC) in marine environments and contribute to several processes, such as particle formation, sedimentation and cycling of dissolved metals [5,6].

**Figure 1:**
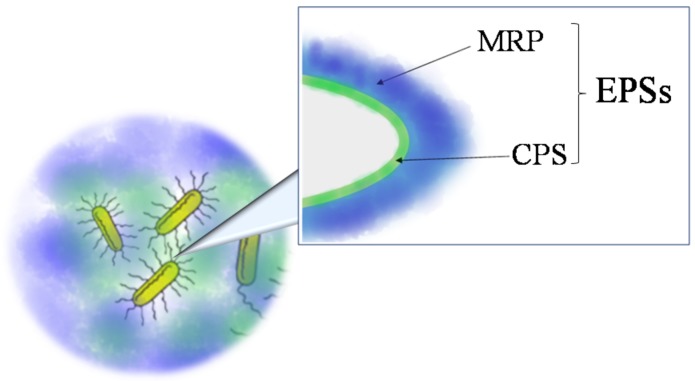
Schematic picture illustrating the typology of exopolysaccharides (EPSs): medium released exopolysaccharides (MRPs) and capsular polysaccharides (CPSs).

Exopolysaccharides (EPSs (Sometimes the acronym EPS is used for ‘extracellular polymeric substances’ to indicate several organic compounds (polysaccharides, nucleic acids, proteins, lipids, etc.) secrete from microorganisms into their surrounding environment. In this review, EPS indicates only exopolysaccharides.)) are glycopolymers that microorganisms secrete in their surrounding environment. They include capsular polysaccharides (in this review named CPSs), if polymers are strictly associated to cell membrane and medium released exopolysaccharides (in this review named MRPs) (Figure 1). These last can be found in the surrounding liquid medium or embedded into biofilms.

The growing interest in renewable sources situates exopolysaccharides as important products of microbial biotechnologies aimed to convert discarded materials into bioenergy and biomaterials, in order to contribute to a reduction in economic dependence on fossil fuels [7].

To this aim, scientists have addressed their attention to oceans, which cover 71% of the Earth’s surface, with an average depth of 3.8 km, an average pressure of 38 MPa (corresponding to 375 atm) and an average temperature of 2 °C. These features cause the existence of various extreme habitats in marine ecosystem, including deep-sea, hydrothermal vents, volcanic and hydrothermal marine areas, marine salterns and salts lake and sea ice in polar regions, niches where extremophilic bacteria thrive and can virtually produce peculiar exopolysaccharide structures.

Many reviews have been already published about exopolysaccharides from marine bacteria and marine extremophiles [8,9,10,11,12,13,14,15,16,17,18]. They deal with bacterial growth conditions, genomic profile and/or polysaccharide properties, biological activity and applications. Instead, very few at our knowledge, discuss on the possible relationships between the detailed chemical structure of exopolysaccharides and their properties. The lack of this type of review depends mainly on the high complexity of exopolysaccharide structures and last but not least, on the difficulty to ascertain the polysaccharide purity. As consequence, the usual chemical characterization of exopolysaccharides based on monosaccharide composition could not have any value for the relationships with properties or activities of exopolysaccharides, without a full polysaccharide structure determination. The development of analytical purification techniques and the sophisticated ^1^H- and ^13^C-NMR 1-D and 2-D sequences approaches allow nowadays to obtain sound detailed polysaccharide primary structures and, in rare cases, conformational investigations, which are important to get information on higher structures.

Aim of this review is to collect the majority of papers containing structural characterization focusing on relationships with polysaccharide activities or properties. A better understanding of the structure property relationships of marine bacteria EPSs is important to evaluate their ecological roles and for exploring their possible biotechnological and industrial applications.

## 2. Purification and Characterization Methodologies

**Figure 2:**
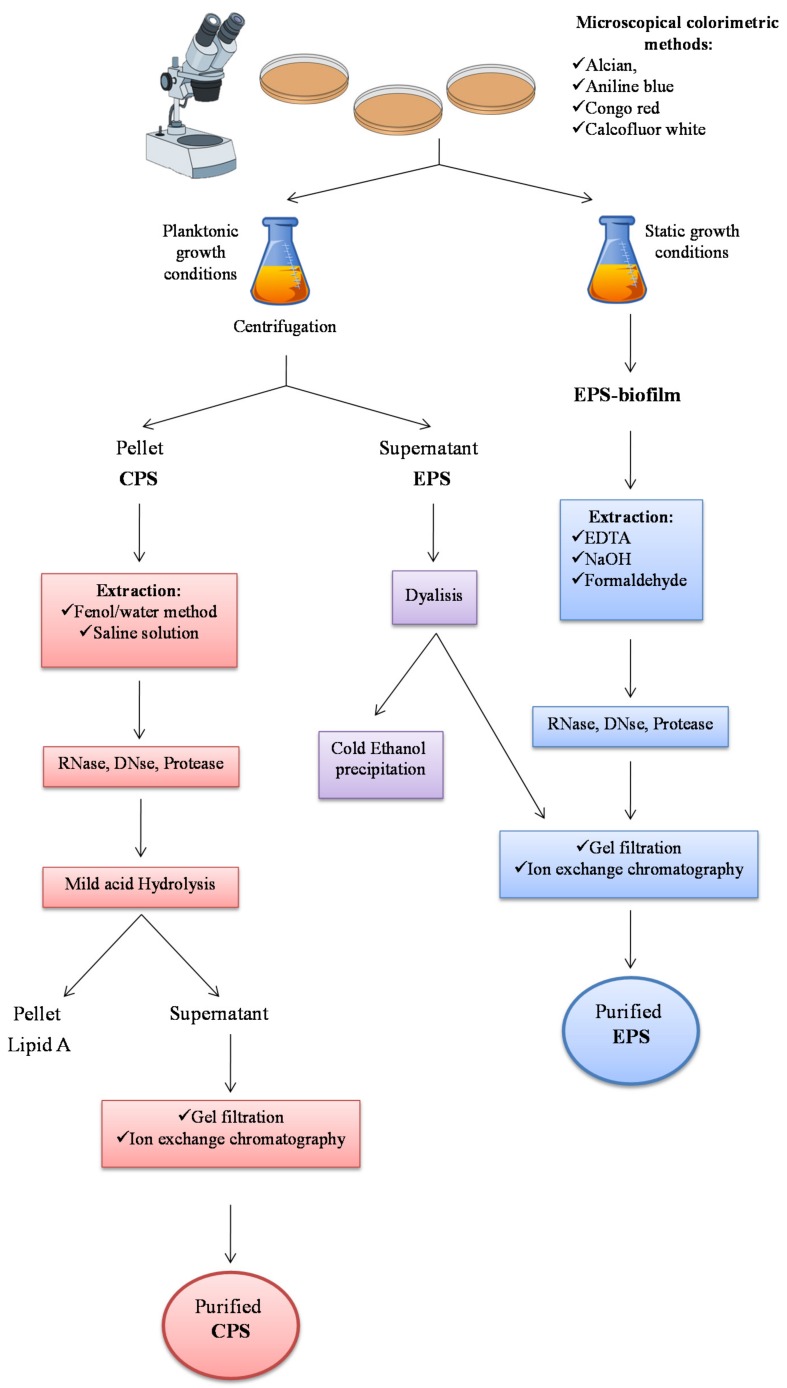
General scheme illustrating detection, extraction and purification of exopolysaccharides from microorganism’s planktonic and sessile growth.

This section focuses on purification and characterization methods targeting marine exopolysaccharides. Firstly, general EPS visualization methods are described, followed by specific descriptions of isolation and purification depending on the polymer localization (Figure 2).

Visualization of polysaccharide material during the microbial growth on agar plates can be reached through colorimetric methods. It is well known that some monosaccharides are able to interact with dyes and this phenomenon can be used to quickly and easily identify the presence of EPS [19]. Among these dyes, Alcian Blue is sensitive to the presence of negative charges of the polymer [8,20], while Aniline Blue [21], Congo Red [22] and Calcofluor White [23] are able to detect glucan polysaccharides.

The electrophoresis is the optimal technique for screening the presence of ionic polysaccharides as well as for following the purification during the various steps. Electrophoresis on polyacrylamide gels (SDS- or DOC-PAGE) of carbohydrates containing molecules is familiar to people working on polysaccharides structural elucidation [24]. Indeed, lipopolysaccharides (LPSs), proteoglycans and charged polysaccharides can be analysed with this technique. Two different staining methods can be used, the silver staining [25] and the Alcian Blue [26,27]. The last one needs the presence of negative charges to be effective.

Exopolysaccharides can be also identified by bacteria phenotypes, both in liquid and solid media. Usually, a “mucoid” strain indicates that the microorganism is able to produce exopolymeric substances but only the determination of carbohydrates content can ensure the presence of exopolysaccharides [28,29]. A number of colorimetric assays has been developed for detecting carbohydrates in a sample. Most of them rely on the action of concentrated (or near concentrated) sulphuric acid, causing the hydrolysis of all glycosidic linkages with a resulting dehydration of the monosaccharides, producing derivatives of furfural. These products react with several reagents, such as phenol [30], *m*-hydroxy-diphenyl [31] and carbazole [32], to give coloured compounds.

EPSs can be extracted by a plethora of physicochemical methods. All of them are able to use external forces to ensure the complete detachment of the polymer from the cells or the complete recovery from the medium. Centrifugation, filtration, dialysis, precipitation, alkaline extraction, metals complexation by EDTA or crown ether and ultrasonication are among the most used methods. It is important to underline that care should be taken when using such methods, in order to avoid cells lysis. However, the extent of cells lysis during these procedures is difficult to evaluate [33].

### 2.1. Extraction from Cell-bound Polysaccharides: Capsular Polysaccharides (CPSs)

Exopolysaccharide isolation is different depending on the polymer localization. Capsular polysaccharides, visualized by Transmission Electron Microscopy [34] and/or light microscopy [35], are mainly recovered from the cells (Figure 2). Indeed, they are strictly associated to the membrane and form ionic interactions with the molecules included in the membrane. The first step consists in the separation of cells from growth medium by centrifugation. During this operation, some CPS molecules could pass into the growth medium, distributing the polymers into the two phases. It turns out that the polymer yield calculation is quite complex.

Then, the isolation of capsular polysaccharides proceeds with a solvent extraction of the cells, usually by saline solution [36] or by the hot phenol/water method [37,38]. Since with the last procedure LPSs are extracted from the cells together with the CPSs, an acetic acid hydrolysis is demanding to remove the glycolipid portion (lipid A) from the polysaccharide sample. After extraction and taking into consideration the sample characteristics, anionic exchange chromatography and/or gel filtration are usually performed.

### 2.2. Extraction from Growth Medium: Medium-released Polysaccharides (MRPs)

MRP-EPSs can be obtained from the growth medium, after centrifugation or filtration of the cells. Then, the supernatant is treated with various chemical substances in order to obtain a precipitate (Figure 2). The majority of the reports indicate alcohol precipitation as the main method for obtaining polysaccharidic material, either by ethanol or 2-propanol. In addition, acetone [39] or detergent salts such as the cetylpyridinium chloride [40], can be used. However, since it is hard to directly obtain a purified polysaccharide, the chromatographic step is determining.

### 2.3. Extraction of EPS from Biofilm

Extraction of polysaccharides from biofilm is much more difficult, due to the necessity to firstly break down the interactions between the EPSs and the complex matrix, mainly constituted by proteins and nucleic acids, where they are embedded [41]. EPSs can be extracted from biofilm by EDTA [42,43,44], NaOH [43], NaCl [45] and formaldehyde [44]. EPSs can also be extracted by centrifugation, as reported for *Pseudomonas aeruginosa* [46]. After extraction and precipitation by ethanol, chromatographies are requested to obtain a pure sample of EPS.

### 2.4. Methods for Structural Characterization

**Figure 3:**
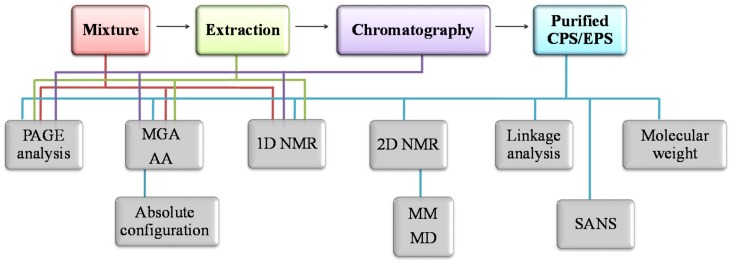
General flow diagram for the characterization of polysaccharide primary and secondary structures performed during each step of purification.

To solve the complete primary structure of an oligo-/polysaccharidic chain the following questions must be addressed: (i) the monosaccharide composition, with their absolute configuration; (ii) the anomeric configurations of the monosaccharides; (iii) the ring size of each monosaccharide; (iv) the glycosylation sites; (v) the sequence of the monosaccharides; (vi) the presence and the positions of the appended groups (Figure 3).

Current analytical methods to characterize a polysaccharidic chain exploit both chemical and spectroscopic methods. Glycosyl analysis is usually performed to establish the type and the relative amount of the monosaccharides constituting the investigated polymer. Chemical derivatization of monosaccharides is necessary to perform such analysis by Gas Chromatography Mass Spectrometry (GC-MS). Therefore, a portion of the polysaccharide is usually subjected to a complete degradation with acids, with two different procedures, owing to the different stability of some monosaccharides. The sample can be either hydrolysed in water and treated with dry acid methanol in a solvolysis reaction (methanolysis). After neutralization and concentration, the sugars are converted into their alditol acetates (AA) [47] or acetylated methyl glycosides (MGA), respectively [48]. The components are separated into the GC column and identified by their relative retention times and Electron Ionization-Mass Spectrometry (EI-MS) fragmentation patterns [49].

For the determination of absolute d or l configurations, the solvolysis is performed with an optical chiral pure alcohol, such as 2-(+)-butanol or 2-(+)-octanol [50]. The diastereoisomeric butyl- or octyl-glycosides obtained, can be acetylated and analysed by GC-MS and the configuration can be deduced by comparison with authentic standards.

The determination of the ring size and the glycosylation sites of the monosaccharides is obtained through the analysis of the partially methylated alditol acetates. These are achieved by a complete methylation of the polysaccharide in alkaline conditions, followed by the acid hydrolysis, the reduction with a deuterated reagent of anomeric positions and acetylation [51]. The observed fragments in MS spectra of GC-MS chromatograms are diagnostic for the specific substitution pattern of methyl and acetyl groups.

Spectroscopic methods include Infrared Spectroscopy (IR) and Nuclear Magnetic Resonance (NMR). FT-IR can furnish several information about polysaccharide structures, such as the type of sugar rings, the presence of amino sugars, uronic acids and sulphate groups. Nevertheless, infrared spectroscopy is not suitable for an in-depth structural analysis.

Instead, Nuclear Magnetic Resonance provides the most useful tool in the field of structural determination of polysaccharides. Anomeric resonances in both ^1^H and ^13^C spectra are usually displayed in a different region with respect to the carbinolic signals, thus helping in determining not only the alpha or beta anomeric configuration of each single residue but also in establishing, in a quite confident way, the number and their relative proportions [52]. Finally, the observation of long-range scalar couplings between anomeric signals and proton/carbon resonances of attachment points in modern 2-D techniques (HMBC), together with the measurement of nuclear Overhauser effects (NOE) between protons two consecutive residues, give information about the sequence. Mono- and two-dimensional spectra of both ^1^H and ^13^C nuclei provide essential and definitive information on the primary structure of a carbohydrate chain. The detailed use of NMR in carbohydrates structural elucidation have been extensively reviewed in dedicated papers [53,54,55,56].

All these methods allow the determination of the primary structure of EPSs, that is only the first step to understand the relationship between the polysaccharidic structure and its function and/or activity. Therefore, secondary structure definition, that is the conformation of the polymer in a solution and in some cases, supramolecular structures characterization, are mandatory to define the shape of the macromolecules in solution.

Among the methods used for secondary structure determination, NMR NOEs measurement coupled with Molecular Mechanics (MM) and Molecular Dynamics (MD) simulations [57], Circular Dichroism [58] and Small-Angle Neutron Scattering (SANS) [59], are the most used.

## 3. Structure of Exopolysaccharides (EPSs) from Marine Bacteria and Archaebacteria

In the following sections, the structural features of EPSs produced by marine bacteria will be reported. The reported EPSs come from species belonging to both Archaea and Bacteria domains. Since many bacteria are ubiquitous, in this review we have considered only marine strains.

### 3.1. Alteromonas

The Gram-negative genus *Alteromonas*, belonging to the Alteromonadaceae family, was established by Baumann and co-workers [60] for marine Gram-negative heterotrophic bacteria requiring sodium to grow [61]. Several species belong to this genus and *A. macleodii* was designated as the type species [60]. Numerous rod-shaped bacteria, which produced EPS, were isolated from alvinellids: *Alteromonas macleodii* sub. fijiensis biovar deepsane HYD 657, *Alteromonas HYD-1545* and *Alteromonas* sp. strain 1644.

*Alteromonas macleodii* subsp. fijiensis biovar deepsane HYD 657 [62,63] produces a high molecular weight exopolysaccharide, named deepsane. This EPS was found to contain different type of sugars carrying sulphate, lactate and pyruvate substituents (Table 2). Deepsane is currently used in cosmetics due to its protective effect on keratinocytes.

The exopolysaccharide produced by *Alteromonas* HYD-1545 was composed of uronic acids and neutral sugars, of which, one galactose unit is substituted by a pyruvic group. This polysaccharide showed a good heavy-metal-binding capability, providing in this way protection to the polychete worm [64].

**Figure 4:**
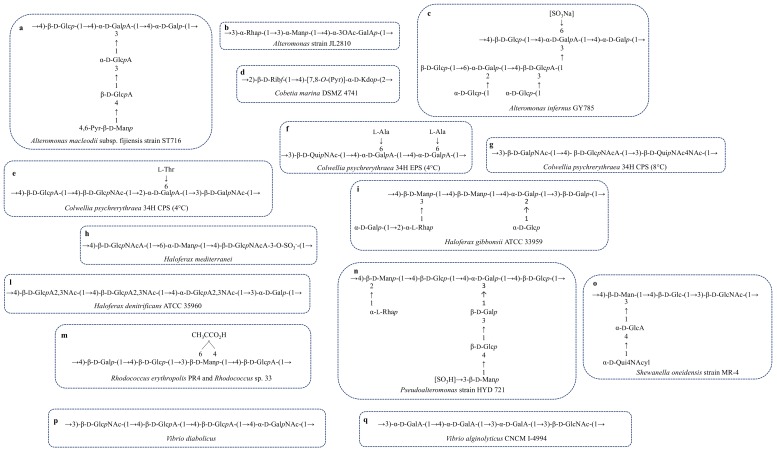
Repeating units of EPS and CPS structures from marine bacteria and archaebacteria. (**a**) *Alteromonas macleodii* subsp. fijiensis strain ST716; (**b**) *Alteromonas* strain Jl2810; (**c**) *Alteromonas infernus* GY 785; (**d**) *Cobetia marina* DSMZ 4741; (**e**) *Colwellia psychrerythraea* 34H CPS (4 °C); (**f**) *Colwellia psychrerythraea* 34H EPS (4 °C); (**g**) *Colwellia psychrerythraea* 34H CPS (8 °C); (**h**) *Haloferax mediterranei*; (**i**) *Haloferax gibbonsi* ATCC 33959; (**l**) *Haloferax denitrificans* ATCC 35960; (**m**) *Rhodococcus erythropolis* PR4 and *Rhodococcus* sp. 33; (**n**) *Pseudoalteromonas* strain HYD 721; (**o**) *Shewanella oneidensis* MR-4; (**p**) *Vibrio diabolicus* (**m**); (**q**) *Vibrio alginolyticus* CNCM I-4994.

The deep-sea bacterium *Alteromonas macleodii* subsp. fijiensis strain ST716 produced an EPS with a good intrinsic viscosity similar to the xanthan’s [65]. The polymer was constituted by a branched hexasaccharide repetitive unit containing Glc, GlcA, Gal and GalA and terminating with a 4,6-*O*-(1-carboxyethylidene)-Man*p* (Figure 4a, Table 2) [66].

*Alteromonas* sp. JL2810 was isolated from the surface seawater of the South China [67]. The exopolysaccharide produced was purified from the culture medium by adding cold ethanol, followed by an anion-exchange chromatography. NMR analysis of the pure EPS showed a trisaccharide repeating unit containing rhamnose, mannose and galacturonic acid (Figure 4b, Table 2). JL2810 EPS showed high capacity to absorb heavy metals (Cu^2+^, Ni^2+^ and Cr^6+^) [68].

Raguènés et al. [69] reported EPS structural features produced by *Alteromonas infernus* strain GY785. This bacterium, isolated near an active hydrothermal vent, produces two different polysaccharides: a water-soluble EPS (EPS1) and a not-soluble EPS (EPS 2) embedded in a gelatinous matrix containing also bacterial cells. Chemical analysis, performed only for EPS1, revealed an acidic polysaccharide containing galactose, glucose, galacturonic and glucuronic acids (Figure 4c, Table 2) [69]. An in-depth characterization of the polymer was achieved by studying oligosaccharide fractions obtained from an acidic hydrolysis. The polysaccharide structure consists of a nonasaccharide repeating unit decorated with sulphate groups [70]. The high content of uronic acids correlated to the metal binding ability and it makes the polymer a good candidate in the waste-water treatment and metal recovery.

*Alteromonas hispanica* F32, a moderate halophilic bacterium isolated from hypersaline habitat in Spain, produces an exopolysaccharide when grown in MY medium supplemented with 7.5% (*w*/*v*) of sea-salts. The molecular weight reported was 1.9 × 10^7^ Da, the highest reported for this type of microorganism. Chemical analysis revealed the presence of Glc, Man, Rha and Xyl as monosaccharide components, together with sulphate and phosphate groups (Table 2). *A. hispanica* EPS showed to be a more efficient emulsifier respect to the commercial surfactants [71].

### 3.2. Bacillus and Geobacillus

The family of Bacillaceae comprises the rod-shaped bacteria which form endospores. They are mostly saprophytes, having a wide diversity of physiological characteristics. Their ability to form hardy spores enables them to be widely distributed in nature, from Arctic environments to hot springs and desert sands and from fresh water to salt or marine sediments. Very few reports are available on EPSs production from *Bacillus* and *Geobacillus* genera. Usually, genus *Geobacillus* comprises a group of Gram-positive thermophilic bacteria that can grow over a range of 45–75 °C.

*Geobacillus* strain 4004 isolated from geothermal sea sand in Italy (Ischia Island) and able to grow at 60 °C as optimal temperature, released an exopolysaccharide in the culture medium. The EPS fraction, recovered by cold-ethanol precipitation, was further purified and three different EPS fractions (named EPS 1–3) were obtained (Table 2). EPS 1 and EPS 2 contained Man, Glc and Gal; EPS 3 showed the presence of Gal, Man, GlcN and Ara. A preliminary structural characterization was carried out only for EPS 3: the ^1^H and ^13^C NMR spectra revealed that this polymer essentially consists of two sugars having a gluco- and galacto- configurations and three with a manno- configuration. In addition, the molecular weight for EPS was found to be about 1 × 10^6^ Da [82].

*Geobacillus tepidamans* strain V264 was isolated from Velingrad hot spring (Bulgaria) at 79 °C and pH 7.8. The microorganism synthesized an exopolysaccharide showing a glucan-like structure, with a molecular weight higher than 1 × 10^6^ Da (Table 2). The bio-polymer isolated from this bacterium was found to be anti-cytotoxic in brine shrimps [83].

Arena et al. [84] reported the immunomodulatory and antiviral effects of an EPS produced by a strain of *Geobacillus thermodenitrificans*, isolated from a shallow marine vent of Vulcano island, in Italy. The EPS showed a molecular weight of 400 KDa and displayed mannose and glucose as main monosaccharidic components (Table 2) [84].

*Bacillus licheniformis* strain B3-15 is a halophilic and thermotolerant bacterium, isolated from the shallow marine hot springs at Vulcano island (Italy). It is reported to produce an exocellular polysaccharide when grown on kerosene as carbon source [72]. The EPS obtained after ethanol precipitation, was desalted on Sephadex G-50 and further purified on a DEAE-Sepharose CL-6B. The most abundant fraction gave an EPS essentially constituted by mannose (Table 2) [72], with the ability to enhance the metal immobilisation (e.g. cadmium immobilisation). In addition, antiviral and immunomodulatory effects of EPS were evaluated: data suggested that EPS treatment impaired HSV-2 replication in human peripheral blood mononuclear cells (PBMC) [73].

Still from Porto Levante (Vulcano, Italy), another thermophilic *Bacillus* strain B3-72 was found to be able to produce two exocellular polysaccharides (EPS1 and EPS2) (Table 2). EPS1 and EPS2 structures were based on mannose and glucose in a relative ratio of 0.3:1 and 1:0.2, respectively [74].

The haloalkalophilic bacterium *Bacillus* sp. I-450, recovered from mudflats (Korea), displayed interesting flocculating activity. The polysaccharide consisted of neutral sugars such as glucose, galactose and fructose (Table 2). The polymer, observed by SEM microscopy, showed a porous surface, where the water molecules are entrapped. In addition, the small pores structure may also be responsible of the polymer compactness, of the gel stability when subjected to external forces and of the maintenance of the texture properties during storage [75,76].

### 3.3. Colwellia

The genus *Colwellia* comprises heterotrophic and halophilic Gammaproteobacteria. It contains 12 psychrophilic species [128,129] and among them *Colwellia psychrerythraea* 34H (*Cp*34H) is the one best characterized [130,131,132]. *Cp*34H was isolated from enriched Arctic marine sediments at −1 °C and it is considered a model organism for cold-adaptation mechanisms [133]. This strain, when grown in marine broth at 4 °C, is able to produce cryoprotectant carbohydrate polymers, an EPS [78,79] and a CPS [37]. In addition, it has been reported that these polymers can be useful for overcoming threshold requirements for dissolved organic carbon in cold environments [131].

The repeating unit of both CPS and EPS have been completely delucidated and both contain amino sugars and uronic acids (Figure 4e,f, Table 2). Another common feature is the presence of an amino acidic decoration, consisting of a threonine and an alanine in CPS and EPS, respectively. The shape of these two polymers in aqueous solution can explain the ice recrystallization inhibition (IRI) activity, measured for both molecules.

When *Colwellia* is grown at 8 °C, it is able to produce a third polysaccharide, which is not endowed with antifreeze activity. This is a polymer containing amino sugars and aminouronic acids and it is not decorated by amino acids (Figure 4g, Table 2) [80]. It has been hypothesized that the lack of these substituents does not settle the equilibrium between the hydrophobic and the hydrophilic regions necessary to the establishment of the IRI activity.

### 3.4. Halomonas

*Halomonas* species are moderately halophilic microorganisms that represent one of the major microbial communities populating marine and saline environments [134]. Several *Halomonas* spp. have been reported as EPS producers, especially those isolated from high salt concentration habitats [92,135].

*H. eurihalina* strain F2-7, previously named *Volcaniella eurihalina*, is a moderately halophilic bacterium isolated from a solar saltern at Alicante. *H. eurihalina* produced an exopolysaccharide in several culture media supplemented with hydrocarbons. Chemical analysis suggested as major components rhamnose, glucose and mannose, with a higher content of uronic acids, acetyl and sulphate groups (Table 2) [87]. EPS from F2-7 resulted a good emulsifier when growth in hydrocarbons media and showed an interesting application in bioremediation of pollutants [88]. Sulphated polysaccharides are quite uncommon among prokaryotic; nevertheless, another species of *Halomonas*, named *maura*, has been reported to produce a sulphated exopolysaccharide, the name of which is mauran. This polymer revealed to be interesting for its potential biotechnological application. Indeed, it showed to share some features with xanthan such as, for example, the ability to form a double helix [89,90]. In addition, solutions of this polymer showed a thixotropic and a pseudo-plastic behaviour, with viscosity not changing in the presence of high salt concentrations or extreme pH values. The monosaccharides composition (Table 2) reflects the xanthan’s, except for the presence of galactose. Finally, the chelating properties of mauran offers the possibility to use it in bio-remediation.

*Halomonas* sp. OKOH was isolated from the bottom sediment of Algoa Bay of South Africa. The bacterium was reported to produce an exopolysaccharide. The phenol-sulphuric acid method revealed a high carbohydrate content, suggesting the polysaccharidic nature of EPS (Table 2). The polymer showed good bioflocculant activity, enhanced in the presence of glucose and urea, used as carbon and nitrogen source, respectively [91].

The EPS released in the growth medium of *Halomonas* sp. AAD6 (JCM 15723) has been found to be a levan [92]. Levans are polymers constituted by β-(2→6)-fructofuranosyl residues (Table 2), with antibacterial [136], anti-cancer and anti-oxidant [137] activities and with probiotic potential [138]. Since levan production is very expensive, an industrial production of this product from *Halomonas* sp. AAD6 has been considered and many efforts have been devoted to obtaining a large-scale production from low-costs substrates [93].

*Halomonas alkaliantarctica* strain CRSS, isolated from salt sediments near the salt lake in Cape Russell in Antarctica, has been shown to produce different EPSs when grown in different conditions [94]. After precipitation of the cell-free supernatant with ethanol, the EPS fraction from each growth medium was further investigated. In all conditions, the glycosyl composition indicated the presence of neutral polysaccharides (Table 2). In particular, it was deduced that CRSS produced on complex media a mannan and a xylo-mannan, whereas on minimal medium, it produced a fructo-glucan [94].

### 3.5. Hyphomonas

Members of *Hyphomonas* genus are Gram-negative bacteria, which are usually colonizer of marine environments. Species of the genus *Hyphomonas* divide by budding, forming a flagellated cell at the tip of the prosthecum [139]. Two marine *Hyphomonas* strains, MHS-3 and VP-6, are reported to produce exopolysaccharides. *Hyphomonas* strains MHS-3, isolated from shallow marine sediments in Puget Sound, produces colonies with different morphology, ranging from a slime-producing (MHS-3) to a non-slime-producing (MHS-3 rad) phenotypes [95]. The EPS produced may be involved in the first phase of adhesion process for the formation of the biofilm matrix.

The capsule-like exopolysaccharide, produced by MHS-3, is recognized by a GalNAc-specific lectin, suggesting that it contains the amino sugar *N*-acetylgalactosamine (Table 2). MHS-3 EPS is an acidic polysaccharide, as confirmed by HPAE and IR analyses and may contain *N*-acetyl-galactosaminuronic acid, due to the binding to the cationic polyferritin [96]. Moreover, it has been reported that MHS-3 EPS has also the ability to sequester gold cations, thus confirming the ionic interactions between the EPS and metal ions [97].

Another prosthecatum bacterium, *Hyphomonas* VP-6, produces two different EPSs, a capsule and holdfast EPS, involved in the first step of biofilm formation (Table 2) [98].

### 3.6. Idiomarina

Mata et al. [71] reported the production of EPSs from two γ-proteobacteria belonging to Alteromonadaceae family, *Idiomarina fontislapidosi* F23 and *Idiomarina ramblicola* R22. 

*Idiomarina fontislapidosi* F23 was isolated from Fuente de Piedra lagoon in Spain [135], while *Idiomarina ramblicola* R22 from Spanish rambla, a steep-sided water course, often dry but subject to flash flooding [135]. Each bacterium produced two different molecular-mass EPSs, mainly composed of Glc, Man, Xyl and Rha, with traces of sulphate and phosphate groups (Table 2). The EPSs produced very stable emulsions, composed of small and uniform droplets, making them good emulsifier agents [71].

### 3.7. Pseudoalteromonas

The genus *Alteromonas*, belonging to the family Alteromonadaceae, was determined by Baumann and co-workers [60] to be a genus of marine Gram-negative heterotrophic bacteria. Later, the genus *Alteromonas* was revised in 1995 to contain only one species, *A. macleodii*, while the remaining species were reclassified as *Pseudoalteromonas* [140,141].

*Pseudoalteromonas* species have been isolated from cold environments (sea-ice, deep sea, Arctic ocean) as well as from marine sea-water. Among these last isolates, there is *Pseudoalteromonas* strain HYD721, a Gram-negative bacterium recovered from a deep-sea hydrothermal vent. The strain, when grown at 25 °C, released a polysaccharide into the growth medium, the structure of which has been completely elucidated [100]. The polymer displays an octasaccharide repeating unit, decorated with a sulphate group (Table 2, Figure 4n). The presence of sulphate groups is quite unusual for *Pseudoalteromonas* genus (marine microorganisms). Sulphated polysaccharides extracted from seaweeds or animals display a huge number of biological functions [142,143]; nevertheless, no assays have been reported for strain HYD721.

Many EPS have found to be involved in metal binding, due to the presence of functional anionic groups, such as carboxyl, phosphoryl, sulfhydryl and hydroxyl groups. These groups can complex both in vivo [144] and in vitro [145] heavy metals, as in the case of the EPS purified from *Pseudoalteromonas* sp. strain TG12, named PE12 [101]. This polymer is a heteropolysaccharide mainly constituted by Glc, GlcN, Xyl and GalA. The sugar analysis revealed also minor components (Table 2). The presence of negative charges of the uronic acids, together with monosaccharides hydroxyl groups has been found to be responsible of the desorption of metals bound to marine sediments [101]. More interestingly is the emulsifying activity of PE12, able to produce stable emulsions with various food oils even at very low concentration (0.02%). An anti-biofilm activity was revealed for a pool of polysaccharides isolated from *Pseudoalteromonas ulvae* strain TC14 grown in sessile conditions [102]. These polymers—named LB-EPS and TB-EPS depending on whether they are loosely or tightly bound to the cells—and the medium soluble EPS (Sol-EPS), inhibit the biofilm of some marine bacterial strains. Their monosaccharide composition was similar, since all of them showed to contain glucose, while only for LB-EPS and TB-EPS an uronic acids content was revealed (Table 2). After various purification steps, TB-EPS was found to be composed of two polysaccharides, of which one is an α-glucan, as suggested by its ^1^H NMR spectrum, while the other is an heteropolysaccharide. A comparison of *P. ulvae* polysaccharides production grown in planktonic conditions revealed a higher content of the Sol-EPS.

*Pseudoalteromonas ruthenica*, isolated from the sea water samples collected from the vicinity of the Bay of Bengal on the coast of India, is able to produce an EPS with interesting properties for potential industrial application [103]. Indeed, the polymer solutions indicated a non-Newtonian behaviour, revealing that it is pseudoplastic in nature. GC-MS analysis revealed that the polymer was mainly constituted by mannose, with a low content of uronic acids (Table 2). The last EPS isolated from a non extremophilic *Pseudoalteromonas* is the one produced by the strain MD12-642. Again, the authors report the capacity of the polymer to confer viscosity to the medium, which suggests potential applications [104]. The galacturonic acid is the most abundant monosaccharide (44%), followed by glucuronic acid (28%), rhamnose (15%) and glucosamine (14%).

*Pseudoalteromonas* genus is frequently found in cold environments. Among these, there are isolates from sea-ice, Antarctic sea-water, Arctic ocean and deep-sea. The cold-adapted *Pseudoalteromonas haloplanktis* TAC125, isolated from Antarctic sea water, is a Gram-negative bacterium, the genome of which has been annotated in 2005 [146]. It is able to grow even at sub-zero temperatures and it has been considered a good model for the cold adaptation [146]. The production of EPS for this microorganism has been reported for three different grown temperatures, namely 4, 15 and 25 °C [105]. In all the cases, the medium contained both carbohydrates and proteins and the purification afforded a phosphomannan structure (Table 2), as indicated by NMR spectra and chemical analysis. Some differences were displayed for the EPS structures obtained at the three different temperatures. In particular, a different phosphorylation content was found, being that of EPS recovered at 25 °C the highest (4.1%), followed by 4 °C (1.4%) and 15 °C (1.1%). In addition, the sample obtained at 15 °C was less branched, indicating a more linear structure [105].

A mannan structure was also recovered from the medium of *Pseudoalteromonas* strain SM 20310, an Arctic sea-ice isolate [106]. This microorganism was selected among 110 isolates obtained from samples collected for cold-active enzyme study. It is well known that the organic matter in marine environments is constituted by high molecular weight polymers produced by microorganisms [147]. These polymers, which in a large amount are polysaccharides, may have different roles, among which is the cryoprotection. The mannan from the strain SM 20310 has been tested for a cryoprotection activity during freeze-thaw cycles of both *E. coli* and the strain itself cells (Table 2). The results gave indications about a significant improvement of the freeze-thaw survival ratio of both *E. coli* and strain 20310, thereby suggesting that this EPS may have a universal impact on microorganism cryoprotection [106]. Moreover, Liu et al. [106] demonstrated that the EPS could enhance the high-salinity tolerance of SM20310, thus facilitating the adaptation of the strain to the sea ice environment.

A cryoprotection role was also found for the exopolysaccharides purified from the two species, *Pseudoalteromonas arctica* KOPRI 21653 [107] and *Pseudoalteromonas elyakovii* Arcpo 15 [40]. The EPS purification method was the same for both strains, even if the biochemical characteristics of the two exopolysaccharides were completely different. Indeed, the glycosyl composition of the arctica polymer revealed only neutral sugar, whereas that of *elyakovii* species indicated also uronic acids (Table 2). As both neutral and acidic EPS have been found to display cryoprotection to microorganism cells, it is hard to deduce how the glycosyl composition can influence the activity.

EPS from marine isolates also play a role in the attachment to surfaces [11], driving marine particle formation including marine microgels, marine snow and biofilms. Hydrophobic interactions could be responsible of cells aggregation and therefore the sugar composition of the EPS, together with secreted proteins, can influence this phenomenon [6]. Two *Pseudoalteromonas* strains, named CAM 025 and CAM 036, were isolated from particles collected in melted Antarctic sea ice and from particles captured by a plankton net towed through the Ocean, respectively. Both the EPSs isolated contain uronic acids, as well as amino sugars, neutral sugars and sulphate groups (Table 2) [108]. The negative charges, due to both carboxylic and sulphate groups, are suggested to be responsible of the “sticky” quality of the EPS in marine environment. In addition, Mancuso-Nichols et al. [148] speculated on the possibility that metal ions, such as dissolved iron, interact with organic ligands, thus suggesting a further ecological implications of EPS polymers.

A bio-sorption capacity and a flocculation behaviour has been attributed to the EPS isolated from *Pseudoalteromonas* strain SM 9913 [109]. This polymer, obtained in large quantity (5.25 g/L) under optimal growth conditions (15 °C, 52 h), is also able to bind a wide range of metal cations, suggesting an ecological role in concentrating metal ions to improve the biochemical interactions with the environment [9]. The glycosyl analysis for this EPS indicated mainly glucose, with arabinose, xylose and a minor content of mannose (Table 2). The complete structure was identified by methylation analysis and by NMR spectra and it was found to have a linear arrangement of α-(1→6) linkage of glucose residues with a high grade of acetylation.

The psychrotroph *Pseudoalteromonas* sp. MER144 belongs to a pool of 606 isolates of Antarctic origin and it has been selected on the basis of its mucous growth on agar plates [110]. To obtain the highest production of the EPS material, many variables were tested, such as different values of carbon source, temperature, pH, NaCl concentration and incubation time. The optimal growth was reached at 4 °C, at pH 7, with a 3% of NaCl, using sucrose as carbon source. After purification, the EPS material showed to still contain proteins, together with a polysaccharide containing neutral sugars and uronic acids (Table 2). Caruso et al. [110] demonstrated that in presence of heavy metals *Pseudoalteromonas* sp. MER 144 is able to increase the production of EPS, suggesting that this could represent an adaptation mechanism to the tested stressed conditions. 

### 3.8. Pseudomonas

*Pseudomonas* species are widespread in marine ecosystems and therefore many EPSs from this genus have been isolated, characterised and tested for their properties and abilities. One of the first species reported to produce exopolysaccharides involved in adhesion properties of the bacterium is the strain NCMB 2021. Two different polysaccharides have been isolated from this strain, of which polysaccharide A was able to form gel, with the ability to bind multivalent cations [111], in contrast with polysaccharide B which is not able to precipitate in the presence of cations. These properties may be related to the glycosyl composition (Table 2), which is completely different in the two polymers (Table 2). Indeed, the solution of polysaccharide B showed low viscosity, suggesting that it has a very flexible chain (random coil). Another EPS involved in the adhesion properties of bacterial cells is that from *Pseudomonas* sp. S9. Under laboratory conditions, the cells were grown in static and, after 1 to 5 h of starvation, they were detected to be surrounded by an exopolymer material [112]. In this case, the cells did not show adhesion to hydrophobic surfaces. With extended starvation, the cells appeared to gradually lose the extracellular layer of polysaccharides. When the cells were grown under agitated conditions, the adhesion characteristic grew between 1 and 4 h of starvation, followed by a slow decrease in attachment with time. In this case, no exopolymer was observed around the cells. Three neutral sugars were revealed for the purified exopolysaccharide (Table 2), even if no relationships with adhesion properties of the cells were suggested.

Sulphated exopolysaccharides are usually isolated from algae and, due to their similarity with glycosaminoglycans, very often display biological activity, anticoagulant, antiviral and immuno-inflammatory activities that might find relevance in nutraceutical/functional food, cosmetic/cosmeceutical and pharmaceutical applications [149]. An anti-cancer sulphated polysaccharide has been isolated and characterised from *Pseudomonas* sp. WAK1 [113]. The structure, obtained by methylation analysis and both mono- and two-dimensional NMR experiments, indicated a backbone of two sulphated galactoses, of which one is branched at 6 positions with a glucose unit (Table 2) [113]. This polymer, showing a cytotoxic effect towards human cancer cell lines MT-4, could provide an example of bacterial exopolysaccharide with suitable functions for obtaining new drugs. Among the most interesting bioactive polysaccharides isolated from *Pseudomonas* species there is the recently reported polymer from *P. stutzeri* 273, which showed anti-biofilm, anti-biofouling and antioxidant activities (Table 2) [114]. All these properties indicated that it could be find applications in challenging bacterial biofilm-associated infection, food-processing contamination and marine biofouling. The glycosyl analysis indicated only neutral and amino sugars, even if the presence of carboxylic acids was suggested by the IR spectrum [114]. Therefore, an in-depth analysis of the primary and secondary structures of this polysaccharide would be interesting in order to advance knowledge of antibiofilm activity mechanism.

A cryoprotectant polysaccharide, containing both neutral monosaccharides and uronic acids, has been isolated and purified from the Antarctic cold-adapted *Pseudomonas* sp ID1 cells (Table 2). Interestingly, the chemical analysis revealed also the presence of amino acids, which could be part of the EPS [115], a feature revealed also for *Colwellia psychrerythraea* 34H CPS and EPS [37,79]. In addition, the polysaccharide was revealed to possess the ability to form a long-term stable emulsion against different food and cosmetic oils, such as olive, sunflower and corn oils and cetiol V oil, respectively [115].

### 3.9. Rhodococcus

*Rhodococcus* sp. 33, a marine bacterium of genus *Rhodococcus*, was isolated from Port Botany (Sidney), in a contaminated site near a chemical plant [150]. This bacterium is able to tolerate and degrade highs levels of benzene. Aizawa et al. [116] reported a different benzene-tolerance for rough and mucoid *Rhodococcus* sp. 33 cells. They demonstrated that the spontaneous mutants rough-type, producing very low amount of EPS, were more sensitive to benzene, resulting in an absent or reduced growth in the presence of the pollutant. In a different way, wild-type colonies that produced EPS appeared mucoid and they were resistant to benzene. These data suggested the direct involvement of exopolysaccharides in the protection against this pollutant [116]. The EPS, purified through an enzymatic digestion and gel filtration chromatography, was analysed by way of chemical and spectroscopic experiments. The polymer consists of a tetrasaccaridic repeating unit containing Glc, Gal, GlcA and Man substituted by a pyruvic acid (Table 2, Figure 4m). Authors demonstrated that the pyruvic residue and the uronic carboxyl group were responsible for the protecting activity, since the de-pyruvylated and carboxyl-reduced EPS that tested for benzene sensitivity showed no activity [117].

*Rhodococcus erythropolis* PR4 was isolated from Pacific Ocean, in Japan. As reported for other strains [151,152,153,154], *R. erythropolis* produces a FACEPS, a fatty acid containing exopolysaccharide. *R. erythropolis* was grown on IB agar plates at 25°C; the EPS purified through an ion exchange chromatography, showed two peaks, named FR1 and FR2, displaying different monosaccharide composition and emulsifying activity. EPS FR1 contained Glc, GlcN, Man and GlcA and did not show any emulsifying activity. Differently, EPS FR2 showed good activity, probably related to the different chemical structure. Indeed, it displays a tetrasaccharidic repeating unit containing Gal, Glc, Man and GlcA and a pyruvic acid substituting the mannose residue (Table 2, Figure 4m). Furthermore, only FR2 EPS contained stearic and palmitic acids. These data allowed the conclusion that EPS FR2—named PR4—was the FACEPS, while FR1 was assigned as a mucoidan (Table 2) [118].

### 3.10. Shewanella

The genus *Shewanella* comprises species largely distributed in marine environments, typically isolated from the deep-sea [155,156]. They can be both mesophiles and psychrophiles. *Shewanella* genus belongs to Gammaproteobacteria and about 60 species have been isolated and characterised. Among these, there are *S. onedeinsis* MR-4 [157] and *S. colwelliana* [158,159]. Both species produce capsular polysaccharides. In particular, *S. oneidensis* polymer has been fully characterised, where neutral monosaccharides, amino sugars and uronic acids are included (Table 2, Figure 4o) [38]. The repeating unit has been identified on the basis of chemical analysis and NMR experiments. Instead, *S. colwelliana* capsule has been characterised by cross-reaction with six monoclonal anti-bodies [120], indicating a specific and identical epitope recognition. Unfortunately, no structure has been identified in this case, only glycosyl analysis and pyruvate content have been reported (Table 2) [120].

### 3.11. Vibrio

The genus *Vibrio* consists of more than 100 species that occur naturally in marine, estuarine and freshwater systems worldwide. They occupy habitats ranging from the deep sea to shallow aquatic environments [160] but are also associated with a wide variety of molluscan and animals, including humans, for some of which are pathogens [161].

Many EPS associated to *Vibrio* bacteria have been reported, of which some are acidic polysaccharides (Table 2). Among these, the only complete structure so far reported for the EPS produced by a polychaete annelid isolate is that from *Vibrio diabolicus*. It consists of a linear tetrasaccharide repeating unit with a structure containing glucuronic acid and rich in amino sugars (Table 2, Figure 4p) [121]. This molecule resembles glycosaminoglycans and it has been tested as enhancers of bone healing. The tests revealed that the polymer was able to act as a filler of bone defects in rat calvaria, with new, well-structured woven bone. They have oriented collagen fibres, with osteoblast cells covering the bone surfaces [122]. In addition, the polymer did not show any inflammatory activity, thus encouraging its application in the healing process.

*Vibrio alginolyticus* is a marine biofouling bacterium, able to produce large quantities of EPS [123]. After purification, the polysaccharide was tested for viscosity measurements. It was revealed to be a pseudoplastic performance, i.e., the viscosity of the solution decreases concomitantly with the shear rate. Authors also reported for the polymer a good stability to pH range between 5 and 10, and the ability to regain its pseudoplastic nature on cooling from 90 °C [123]. The glycosyl composition for the purified polymer indicated only neutral and amino sugars (Table 2). Instead, the EPS isolated from the strain CNCM I-4994 of *V. alginolyticus* was composed of galacturonic and N-acetyl glucosamine monosaccharides (Table 2, Figure 4q) [124]. This polymer is produced on an industrial scale as an ingredient for cosmetic applications for its mattifying and anti-inflammatory properties [124]. The most interesting feature of the repeating unit is the amino acidic decoration, which has also been reported for both *C. psychrerythraea* 34H CPS and EPS [37,79]. This is not surprising, because *Vibrio* and *Colwellia* are phylogenetically close to each other. Indeed, also the glycolipid portion of the LPS from *C. psychrerythraea* 34H [162] has been found to be similar to that of *Vibrio fisheri* [163].

*Vibrio harveji* strain VB23 [125] and *Vibrio furnissii* strain VB0S3 [126] have both been isolated from the Mandovi and Zuari estuarine in Goa, on the west coast of India. They are both able to produce an acidic exopolysaccharide with emulsifying properties. Unfortunately, only neutral sugar composition has been reported for both polymers, thus hampering any comparison with other *Vibrio* EPSs. An anti-biofilm activity has been reported for *Vibrio* sp. QY101 EPS [127]. This marine bacterium, isolated from a decaying thallus of *Laminaria* [164], was initially supposed to inhibit the growth of *Pseudomonas* for the alginate-lyase activity of the culture supernatant. However, after elimination of the protein content, the anti-biofilm activity was lost. Instead, ion-exchange and gel filtration chromatographies afforded a pure polysaccharide able to inhibit cell aggregates of *Pseudomonas aeruginosa* and *Staphylococcus aureus*. The polysaccharide showed to contain more than 40% of uronic acids and about 10% of GlcNAc, revealing strong similarities with the EPS structures of *alginolyticus* and *diabolicus* species.

### 3.12. Other EPS-Producing Bacteria

*Cobetia marina* (DSMZ 4741), a slightly halophilic Gram-negative bacterium isolated from littoral seawater in USA [165], is often found associated with microalgae. The exopolysaccharide, named L_6_, was produced at 20 °C, pH 7.6 in a fermenter. *Cobetia* EPS, with a molecular weight of 2.7 × 10 ^5^ Da, showed a disaccharide repeating unit containing ribose and a 7,8-pyruvilated Kdo (Table 2, Figure 4d). The presence of Kdo, typically a component of the LPS molecule, has been reported for K-antigen polysaccharide from *Escherichia coli*. The EPS from *Cobetia marina* represented the first example of EPS with this type of structure. The unusual substitution of the Kdo could represent a possible way for the bacterium to escape the viral Kdo-hydrolases, which can be considered a resistance mechanism [77].

Members of the genus *Flavobacterium* are widely distributed in nature, occurring mostly in aquatic environments, from freshwater to seawater [166]. *Flavobacterium uliginosus* MP-55 was isolated from the homogenates of sea weed collected in Japan. The marine bacterium grown in a medium supplemented with marine water produces a water-soluble polysaccharide. The polymer—named marinactan—contained glucose, mannose and fucose (Table 2), with a molecular weight higher than 1 × 10^6^ Da. Biological assays showed that marinactan was an antitumoral molecule against sarcoma 180, in both solid and ascites forms [81].

*Hahella chejuensis* strain 96 CJ10356, a Gram-negative halophilic bacterium, was isolated from marine sediments from Cheju Island (Republic of Korea) [85]. The monosaccharide analysis of EPS-R revealed a heteropolysaccharide consisting of glucose and galactose as main components, with xylose and ribose in minor amounts (Table 2). The molecular weight of EPS-R was approximately 2.2 × 10^3^ KDa. The exopolysaccharide showed a good emulsifier activity [86].

*Polaribacter* sp. SM1127, a member of the genus *Polaribacter*, was isolated from the brown alga *Laminaria* [167]. Bacteria of this genus have been isolated from different marine environments, mainly from Arctic and Antarctic regions. *Polaribacter* sp. SM1127 produces an exopolysaccharide containing Rha, Fuc, Man and Glc, together with a higher amount of GlcA and GlcN. The polysaccharide, with a molecular weight of 220 KDa, showed a good viscosity and antioxidant activity, thus representing a great potential in cosmetics as anti-ageing product. Furthermore, it showed moisture-adsorbing and retention ability superior to that of ialuronic acid and glycerine, both currently used in the cosmetic field. This enhanced activity could be related to the high percentage of GlcA, Fuc and GlcN residues (Table 2). In addition, the cryoprotectant role of EPS was tested on dermal fibroblast at 4 °C. SM1127-EPS, displaying protection to fibroblast cells, could be used as component of cream to protect human skin from cold injury [99].

The bacterium *Salipiger mucosus* A3^T^, belonging to the alfaproteobacterium class, was isolated from a solar saltern on the Spanish Mediterranean seaboard [168]. The production of the EPS from the halophilic *S. mucosus* was observed with all carbon sources tested and the best yield was obtained at a sea-salt concentration of 2.5% (*w*/*v*). The cells, stained with ruthenium red and observed to TEM, revealed a different morphology over the time: at beginning, the polysaccharide appeared strictly associated to the cells, while from the third day of incubation, it was released in the surrounding medium as slime. The purified EPS, showed a molecular weight of 250 KDa and resulted to be composed by Glc, Man, Gal and the uncommon Fuc (Table 2). In addition, the presence of sulphate and phosphate groups make the polysaccharide interesting, since this EPS with high charge density have a great potential for removing toxic metals from environment. *S. mucosus* EPS showed interesting emulsifying activity with several hydrocarbons, compared with other molecules already used as surfactants. Furthermore, it showed a higher emulsifying activity than xanthan with crude oil [119].

At first, bacteria belonging to the genus *Zooglea* were considered members of the Pseudomonadaceae family but later they were differentiated for the production of a gelatinous matrix. Indeed, the name *Zooglea* is derived from Greek and means animal glue, referring to the sticky feature of the zoogloeal matrix [169].

Bacterium *Zooglea* sp. KCCM 100376, isolated from the surface layers of the seaweed *Undaria* sp., was reported to produce two exopolysaccharides: a water-soluble polysaccharide (WSP), recovered in supernatant after centrifugation of growth broth and a cell-bound polysaccharide (CBP) obtained from the precipitate [39].

GLC analysis revealed the same monosaccharide composition (Glc, Gal and Man) for both polysaccharides, even if in different molar ratio (Table 2). In addition, IR spectra of these EPS suggested the uronic acid nature for some sugars. Kwon et al. [39] demonstrated that *Zoogloea* sp. KCCM 100376 could grow and produce polysaccharides without the addition of carbon source, thus showing that most of the glucose added to the medium could be converted to CBP or WSP without increasing the cell mass [39]. The molecular weight, measured by HPSEC, resulted to be 4 × 10^6^ Da and 3.4 × 10^6^ Da for WSP and CBP, respectively. Physicochemical and rheological properties of both EPSs were evaluated. WSP and CBP showed good emulsifying and flocculating activity compared with other commercial products. In addition, the water-holding capacity (WHC) was evaluated for WSP and CBP. The thaw after freezing in food processing determines the loss of moisture and soluble proteins, compromising the integrity of the product. Data indicated that CBP displayed a water-holding capacity higher than xanthan polymer, a common polysaccharide used in food processing and that the activity increased with concentration [170]. The viscosity analysed for both EPSs, was higher for WSP than the CBP's at any concentration assayed. In addition, it showed a good viscosity at high temperature, under pH variation and in presence of salt.

### 3.13. Marine Archaea

Several exopolysaccharides have been isolated from Archaea domain, mainly from microorganisms belonging to thermophilic and halophilic groups. The halophilic genera, *Haloarcula* and *Haloferax*, are reported as the main marine producers of EPS. In addition, thermoacidophilic members of the genera *Thermococcus* and *Sulfolobus* were observed to secrete polysaccharides [171].

Exopolysaccharides production from marine bacteria within the genera *Haloarcula* is reported for three halophilic isolates from the Tunisian marine saltern: *Haloarcula japonica* strains T5–T7 [172]. The exopolysaccharides, produced by the bacteria grown in a minimal medium supplemented with glucose, were precipitated with cold-ethanol, obtaining a good yield only for strain T5 (370 mg·L^−1^ versus 30–40 mg·L^−1^). Sugar composition revealed the presence of Man, Gal and GlcA for *H. japonica* T5 and Man, Gal, Glc and trace of GlcA for *H. japonica* T6 and *H. japonica* T7 strains. No uronic acids were revealed in the EPS from *Haloarcula hispanica* ATCC 33960; instead, only Man, Gal with traces of Glc were found, together with sulphate groups (Table 3) [173].

*Haloferax mediterranei* ATCC 33500 was the first example of archaebacterium able to produce an exopolysaccharide, conferring to the cells a mucous feature. The presence of an EPS surrounding the cells was confirmed by electron microscopy analysis [174]. IR, chemical analysis and NMR spectroscopy allowed to obtain the primary structure, consisting of a highly charged trisaccharide repeating unit containing GlcNAcA and Man and decorated with sulphate groups (Table 3, Figure 4h) [175].

Paramonov et al. [176] reported the EPS structural characterization from another member of the Archaea domain, *Haloferax gibbonsii* ATCC 33959, isolated from marine saltern of the Dead Sea. As for *Haloferax mediterranei*, the mucoid appearance of the colonies in *H. gibbonsii* was attributed to the presence of a polysaccharide. The EPS structure consists of a heptasaccharide repeating unit containing all neutral sugars (Table 3, Figure 4i) [176].

*Haloferax denitrificans* ATCC 35960 isolated from marine saltern in San Francisco Bay, releases in the supernatant an acidic exopolysaccharide. The polymer showed a tetramer made up of three units of the unusual 2,3-diacetamido-2,3-dideoxy-d-glucopyranosiduronic acid (GlcA2,3NAc) and one galactose (Table 3, Figure 4l) [177].

The hyperthermophilic *Thermococcus litoralis* was isolated from a shallow marine thermal spring near Naples, showing an optimal growth temperature of 88 °C. This bacterium produced an exopolysaccharide apparently containing only mannose as monosaccharide constituent (Table 3) [178].

## 4. Structure-Function Relationships and Ecological Role

**Figure 5:**
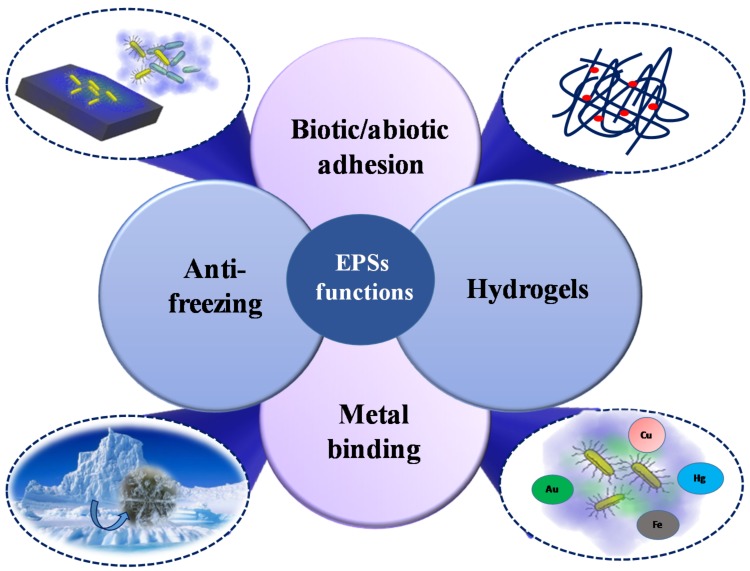
Schematic illustration of some EPS functions.

Marine ecosystems host about the half of the photosynthetic biomass produced on the Earth [3]. All these microbial communities have a role in fundamental processes occurring in marine habitats, responding to environmental changes. Indeed, the climate change—associated shifts in temperature alter the carbon chemistry, the nutrient and the oxygen content and it causes tropical storms and alterations in ocean currents [179]. Since the exopolysaccharides give a huge contribute to biogeochemical cycling in the ocean [6], a deep insight into the ecological function of their production is demanding. Starting from the chemical characterization of these polymers, it might be possible to find relationships between the structures and the numerous functions attributed to them (Figure 5).

Most of the exopolysaccharides showing emulsifying properties include deoxyhexoses, uronic acids and/or fatty acids in their composition (Table 2 and Table 3). These features are shared with other polysaccharidic surfactants, such as Arabic gum, where a subtle balance among these residues is responsible for the molecular conformation and, as a consequence, for the activity [180]. It is noteworthy that, to be a good emulsifying agent, the inclusion of a protein moiety gives a clear-cut improvement to the quality of the emulsifier [180,181]. Therefore, the presence of proteins in most of the exopolysaccharides samples for which a good emulsifying activity has been reported, is not surprising [14].

Negative charges due to uronic acids and acidic substituents, such as phosphates, sulphates and pyruvates, account for the highest percentage of the isolated exopolymers (Table 2 and Table 3). Such exopolysaccharides can be considered polyelectrolytes and therefore are able to form ion cross-linking that are continuously formed and broken, due to the low bond energy (20–40 kJ·mol^−1^) [6]. Na^+^ and Ca^2+^ ions are abundant in marine ecosystems, allowing the formation of cross-linking among EPS chains with the consequent formation of microscopic gels [182]. It has been proposed that such gels are refractory to degradation by bacteria and may constitute a network of nutrient “hot spots” [183]. Bacteria can then attach to gels, using hydrolytic enzymes to give a biochemical process for large scale transfer of organic matter from sinking aggregates to dissolved molecules [184].

Polyanionic EPS may also have a role in controlling Fe (III) bioavailability [185], thus suggesting a significant contribution to the marine organic ligand pool, including siderophores. Indeed, up to now, only few papers describe the quantification of siderophores concentration in the oceans [186,187,188]. Instead, it has been reported that the concentration of Fe-binding ligands associated with EPS compete with that of siderophores, being this last an order of magnitude lower than that reported for EPS [189]. Therefore, it is possible to speculate that most of the here reported EPS structures with a high content of uronic acids and/or negative charged groups (Table 2 and Table 3) contribute to the biogeochemical iron cycle in marine environments.

Capsular polysaccharides are frequently found to be involved in adhesion processes and several EPS/CPS included in this review are reported to be “sticky”. This property is useful for both colonization [107] and for microbial-host interaction [62,63,102]. There is a body of evidence that a huge number of marine microorganisms are able to form biofilms [8,190,191]. The presence and the properties of biofilms are regulated by their components. Among these, EPS have a predominant role in forming a cohesive matrix where cells and other molecules are strictly associated. The colonization of the surfaces occurs through the biofilm formation, as in the case of *Pseudomonas* species S9, isolated from marine sediments, where cells grown in starvation of nutrients were able to enhance the production and the release of EPS. This result translated into a pronounced effect on the degree of adhesion and aggregation of the bacterial cells [141].

The prosthecate bacterium *Hyphomonas* VP-6 is able to produce two different polysaccharides both showing adhesive properties, of which one is a holdfast EPS while the second is a capsule [98]. It has been reported that both are acidic and this feature is responsible for the adhesion process. The structure of the EPS produced by *Hyphomonas* MHS-3 has not been determined yet, even if its involvement in adhesive function of this species has been extensively studied [95]. Unfortunately, for this interesting EPS, only recognition of GlcNAc presence with lectins binding has been reported, thus precluding, at least up to now, any possible structure-function relationship finding.

Since the microbial secretion has been recognized relevant for the biogeochemical cycling in marine environments [11], the variations of the pH towards more acidic values in the oceans could also affect the EPSs production. However, it is not easy to understand the impact of the seawater acidity due to the CO_2_ absorption, because the ions composition and the physical conditions are too complex and variable. Nevertheless, reported data, exploiting the effects of the increased seawater acidity, have been obtained under controlled laboratory conditions. For example, it has been observed that the reduction of calcium carbonate affects shell-forming marine organisms [192] and the microbial-mediated carbon cycle [193]. Interestingly, it has also been reported that the ocean acidification stimulates the bacterial community, facilitating the microbial recycling [194]. Finally, an elevated pressure of CO_2_ has been reported to increase biofilms production as well as the amount of uronic acids in their composition [195] 

**Figure 6:**
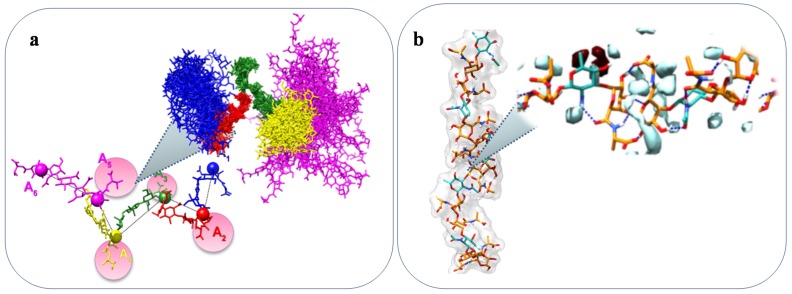
Molecular modelling of *Colwellia psychrerythraea* 34H CPS (**a**) and EPS (**b**).

One example showing a close correlation between structure and a specific property of a marine bacterial polysaccharide, is the anti-freezing activity of EPS and CPS in Arctic and Antarctic bacteria [37,79]. The EPS and CPS presence in Arctic and Antarctic systems with cryoprotectant functions is well documented [20,37,79,111]. In particular, diatoms exopolysaccharides have been described also to attach to sea-ice interfaces and to contribute to the cells survival in sea-ice [20,196,197]. In addition, bacterial exopolysaccharides have been reported to show anti-freeze properties. Indeed, although proteins are by far the most famous polymers with ice-binding properties, very recently two different polysaccharides isolated from *Colwellia psychrerythraea* 34H endowed with anti-freeze activity have been fully described [37,79]. These two polysaccharides are characterized by the presence of amino acidic decoration: the CPS is constituted by a repetitive tetramer resembling glycosaminoglycans (repeating unit with alternating amino sugar and uronic acid), decorated with threonine, whereas the EPS displays a trisaccharidic repeating unit decorated with alanine. These unusual polysaccharides display ice-recrystallization inhibition activity, most probably due to the particular shapes they assume in solution. Indeed, molecular dynamic calculations suggested for the CPS a model displaying a “zigzag” arrangement with threonines occupying external positions, thus resembling some antifreeze glycoproteins (Figure 6a) [37]. As for the EPS, the calculations indicated a left-handed helix, stabilised by a series of inter-residue H-bond interactions. In addition, the calculated tetrahedral order parameter Qk, which measures the propensity of a particular water molecule and its four neighbouring molecules to adopt a tetrahedral arrangement, indicated no tetrahedral geometry of water molecules in the sugar cage formed around the quinovosamine residue (Figure 6b) [79]. 

Another marine bacterium producing cryoprotectant EPS probably decorated with amino acids is that from *Pseudomonas* sp ID1 [144]. It suggests that *Colwellia* polysaccharides may be only two examples of a wider assortment of polymers endowed with ice-binding properties. 

## 5. Biotechnological Applications of Marine EPS 

Polysaccharides are used in several industrial fields, as thickeners, stabilisers and gelling agents in food products and as antitumoral, antioxidant and/or prebiotic in pharmacology [198]. They derived from a variety of sources: bacteria, fungi, algae and plants. Despite all these sources, the world market is dominated by polysaccharides from algae [199], like carrageenans, agar and alginates [200,201] and from lactic acid bacteria, due to the high number of EPSs recovered after the extraction.

In the last years, EPSs produced by marine bacteria have been attracted the interest of several researchers for their unique properties of considerable biotechnological importance and, therefore, of commercial significance. Currently, despite the vast number and biodiversity of the marine EPSs, these polymers represent only a small fraction of the current polymers market, due to the very low amount of purified polysaccharide obtained. Therefore, to achieve a larger amount to be commercialized is necessary to proceed with an enhancement of the production. Particularly, microbial polysaccharides production is greatly influenced by fermentation conditions. Indeed, the structure, the composition and the viscosity of EPSs depend on several factors, such as the composition of the culture medium, the sources of carbon and nitrogen and the precursor molecules, the mineral salts, trace elements, the type of the strain and the fermentation conditions such as pH, temperature, oxygen concentration and agitation [202]. In addition, engineering modifications of genes involved in the polysaccharide biosynthesis could also be convenient.

During the last years, diverse marine microbial exopolysaccharides turned out to be promising candidates in biotechnology field. They span from the exopolysaccharides displaying biological activity, exploitable in the pharmaceutical and medical industry [83,99,102,114,121], with particular regard to the sulphated polysaccharides [62,63,113], to the emulsifier EPSs, that find application in the food, pharmaceutical, cosmetic and petroleum industries [71,85,86,87,118,125,126]. Furthermore, it is important to consider the applications of the cryoprotectant and anti-freezing EPSs in many industrial fields [37,40,79,99,106,107,115].

## 6. Conclusions

From the above data, it is possible to highlight the difficulty to carry out an accurate structural determination of a polysaccharide, which is the preliminary step to establish a relationship with its properties and/or functions. The same study is not possible having only the glycosyl composition information, which, however, are useful to explain some properties. An interesting speculation arising from glycosyl composition regards the land plants grown in saline water. These plants produced polysaccharides containing negative charged groups, as carboxyl groups [203], when grown under salt-stress conditions. Strikingly, marine bacteria seem to have been chosen carboxyl groups, instead of sulphates of sea-weeds, to make their polysaccharides negatively charged, as it results by the common presence of uronic acids in marine EPSs.

In our opinion, this review suggests to further investigate on the structural determination of exopolysaccharides from marine bacteria, both for potential biotechnological applications and to study in depth their biological functions in the biofilm formation. The high difficulty to make this work requires further developments in the polysaccharide purification techniques, microscopic analyses and more sensitive spectroscopic methods. Future developments could be related to the establishment of correlations between the regulation of the genes responsible of exopolysaccharide structures and the conditions of marine environments.

## Figures and Tables

**Table 1:** Typical monosaccharide components of marine EPS.

Monosaccharides	Example
Pentoses	arabinose (Ara), ribose (Rib), xylose (Xyl)
Hexoses	glucose (Glc), galactose (Gal), mannose (Man)
Deoxy-hexoses	quinovose (Qui), fucose (Fuc), rhamnose (Rha)
Uronic acids	glucuronic acid (GlcA), galacturonic acid (GalA), mannuronic acid (ManA)
Amino sugars	glucosamine (GlcN), galactosamine (GalN), mannosamine (ManN)
Uncommon sugars	3-deoxy-d-manno-2-octulosonic acid (Kdo), neuraminic acid (Neu)

**Table 2:** Examples of marine bacterial exopolysaccharides.

Microorganism	Source	EPS Structure or Monosaccharide Composition	Functions and Applications	Reference
***Alteromonas***				
*A. macleodii* sub. fijiensis biovar deepsane HYD 657	Deep-sea hydrothermal vent polychaete annelid	Fuc:Rha:Glc:Gal:Man:GlcA:GalA 1:2.5:2.6:5.9:1.4:2:1.9 Sulphate, lactate and pyruvate groups	Cosmetic, keratinocytes protection	[62,63]
*A*. strain HYD-1545	Hydrothermal vent polychaete annelid	Gal, Glc, GlcA, GalA, GalX 2.5:3:2:2:1 X = pyruvate	-	[64]
*A. macleodii* sub. fijiensis strain ST716	Deep-sea hydrothermal vent	Structure, Figure 4a	Gel forming	[65,66]
*A*. strain JL2810	Sea water	Structure, Figure 4b	Biosorption of heavy metal	[67,68]
*A. infernus* GY785	Hydrothermal vent	Structure, Figure 4c	Metal recover	[69,70]
*A. hispanica* F32	Hypersaline inland	Glc:Man:Rha:Xyl 18:63:7:12 Sulphate and phosphate groups	-	[71]
***Bacillus***				
*B*. strain B3-15 halophile	Marine hot spring	Man	-	[72,73]
*B*. strain B3-72 thermophile	Hydrothermal vent	EPS1, Man:Glc 0.3:1 EPS2, Man:Glc1:0.2	-	[74]
*B*. strain I-450 haloalkaliphile	Mudflats	Glc, Gal, Fru	Gel forming	[75,76]
*Cobetia marina* DSMZ 4741	Coast	Structure, Figure 4d	-	[77]
***Colwellia***				
*C. psychrerythraea* 34H psychrophile	Sea sediments, sea ice	Structure, Figure 4e	Anti-freeze	[37]
*C. psychrerythraea* 34H	Sea sediments, sea ice	Structure, Figure 4f	Anti-freeze	[78,79]
*C. psychrerythraea* 34H	Sea sediments, sea ice	Structure, Figure 4g	No anti-freeze activity	[80]
*Flavobacterium uliginosum* MP-55	Sea weed	Glc:Man:Fuc 7:2:1. Marinactan		[81]
***Geobacillus***				
*G*. strain 4004 thermophile	Sea water	EPS1, Man:Glc:Gal 0.5:1:0.3 EPS2, Man:Glc:Gal 1:0.3:traces EPS3, Gal:Man:GlcN:Ara 1:0.8:0.4:0.2	-	[82]
*G. tepidamans* V264 thermophile	Hot spring	Glucan	Immunomodulant, anti-viral	[83]
*G. thermodenitrificans* thermophile	Shallow marine vent	Man, Glc		[84]
*Hahella chejuensis* strain 96CJ1035	Marine sediments	Glc:Gal 1:6.8 Traces of xylose and ribose	Emulsifier	[85,86]
***Halomonas***				
*H. eurihalina* F2-7	Dead Sea	Glc:Man:Rha 2.9:1.5:1 Uronic acids, amino sugars, sulphate groups	Emulsifier	[87,88]
*H. maura* halophile	Solar saltern	Man:Gal:Glc:GlcA 34.8:14:29.3:21.9 Sulphate groups	Viscous, pseudoplastic	[89,90]
*H*. sp. OKOH halophile	Bottom sediments	No structure	Flocculant	[91]
*H*. sp AAD6 (JCM 15723)	Soil saltern	Levan	-	[92,93]
*H. alkaliantarctica* strain CRSS	Salt lake	EPS1, Mannan EPS2, Xylo-mannan EPS3, Fructo-glucan	-	[94]
***Hyphomonas***				
*H*. strain MHS-3	Shallow marine sediments	GalNAcA	Adhesion	[95,96,97]
*H*. strainVP-6	Vent region	No structure	Adhesion	[98]
***Idiomarina***				
*I. fontislapidosi* F23^T^	Lagoon	Glc, Man, Xyl, Rha Sulphate and phosphate groups	Emulsifier, metal binding	[71]
*I. ramblicola* R22^T^	Water-course	Glc, Man, Xyl, Rha Sulphate and phosphate groups	Emulsifier, metal binding	[71]
*Polaribacter* sp. SM1127 psychrophile	Artic brown alga Laminaria	Rha:Fuc:GlcA:Man:Glc:GlcN 0.8:7.4:21.4:23.4:17.3:1.6:28.0	Cryoprotectant, anti-oxidant	[99]
***Pseudoalteromonas***				
*P*. strain HYD721	Deep-sea hydrothermal vent	Structure, Figure 4n	-	[100]
*P*. strain TG12	Sea-water	Rha:Fuc:Gal:GalNAc:Glc:GlcNAc 1.2:0.5:0.9:0.7:10.4:24.8 Man:Xyl:MurA:GalA:GlcA 4.8:27.7:0.3:23.1:5.6	Metal binding	[101]
*P. ulvae* TC14	Marine biofilm	LB-EPS, Glc, uronic acids TB-EPS, Glc, uronic acids Sol-EPS, Glc	Anti-biofilm	[102]
*P. ruthenica*	Sea-water	Man, traces of uronic acids	Pseudoplastic	[103]
*P*. sp. strain MD12-642	Marine sediments	GalA:GlcA:Rha:GlcNAc 44:28:15:14	Viscosity	[104]
*P. haloplanktis* TAC125 psychrophile	Antarctic sea water	Man, Glc Phosphate groups	-	[105]
*P*. sp. strain SM20310 psychrophile	Arctic sea-ice	Rha:Xyl:Man:Gal:Glc:GalNAc:GlcNAc 2.1:0.9:71.7:9.0:10.7:1.5:4.0	Cryoprotectant	[106]
*P. arctica* KOPRI 21653 psychrophile	Sea-side sediments	Glc:Gal 1.5:1	Cryoprotectant	[107]
*P. elyakovii* Arcpo 15 psychrophile	Not reported	Man:GalA 3.3:1	Cryoprotectant	[40]
*P*. sp. CAM025 psychrophile	Particles form Antarctic sea	Glc:GalA:Rha:Gal 1:0.5:0.1:0.08	Adhesion	[108]
*P*. sp. CAM036 psychrophile	Particles from Southern Ocean	GalA:Glc:Man:GalNAc:Ara 1:0.8:0.84:0.36:0.13	-	[108]
*P*. sp. SM9913 psychrophile	Deep-sea sediment	Glc, Gal, Xyl, Ara	Metal binding	[109]
*P*. sp. MER144 psychrophile	Terra Nova Bay, Ross SeaAntarctic	Glc:Man:GalN:Ara:GlcA:GalA:Gal 1:0.36:0.26:0.06:0.06:0.05:0.03	-	[110]
***Pseudomonas***				
*P*. sp. NCMB 2021	Not reported	Pol A, Glc:Gal:GlcA:GalA 1.45:1.18:0.64:0.43 Pol B, GlcNAc, deoxy-Hex, Kdo	metal binding (A) Adhesion (B)	[111]
*P*. sp. S9 psychrophile	Polar basin	Glc:GlcNAc:GalNAc 28:35:37	Adhesion	[112]
*P*. sp. WAK1	Brown seaweed Undaria pinnatifida	Gal:Glc 2:1 Sulphate groups	Anti-cancer	[113]
*P. stutzeri* 273	Marine sediments	GlcN:Rha:Glc:Man 35.4:28.6:27.2:8.7	Anti-biofilm, anti-biofouling, antioxidant	[114]
*P*. sp. ID1	Marine sediments	Glc:Gal:Fuc 2:1:1	Cryoprotectant	[115]
***Rhodococcus***				
*R*. sp. 33	Contaminate site near a chemical plant	Structure, Figure 4m	-	[116,117]
*R*. erythropolis PR4	Ocean	FR2 Structure, Figure 4m	Emulsifier	[118]
		FR1 Glc:GlcN:GlcA:Fuc 2:1:1:1	-	
*Salipinger mucosus* A3^T^halophile	Solar saltern	Glc:Man:Gal:Fuc 1.5:2.5:2.5:1 Sulphate and phosphate groups	Metal binding, emulsifier, pseudoplastic	[119]
***Shewanella***				
*S. oneidensis* MR-4	Dead Sea	Structure, Figure 4o	-	[38]
*S. colwelliana*	Associate bivalve	Man:Glc:Gal:pyruvate 1:2:2:4	-	[120]
***Vibrio***				
*V. diabolicus*	Deep-Sea hydrothermal ventA. pompejana	Structure, Figure 4p	Filler of bone defects in rat calvaria	[121,122]
*V. alginolyticus*	Sea water	Glc:Xyl:RibN:AraN 2:1:9:1	Shearing properties	[123]
*V. alginolyticus* CNCM I 4994	Sea water	Structure, Figure 4q	-	[124]
*V. harveji* VB23	Sea water	Gal:Glc:Rha:Fuc:Man:Rib:Ara:Xyl 10.08:3.6:0.7:0.15:1.56:0.2:0.3:0.45	Emusilfier	[125]
*V. furnissii* strain VB0S3	Sea water	Gal:Glc:Rha:Fuc:Man:Rib:Ara 5.21:4.68:1.0:0.79:1.43:0.16:0.19	Emusilfier	[126]
*V*. sp. QY101	Laminaria thallus	Glc:Gal:GlcA:GalA:Rha:Fuc:GlcN:Man 6.57:6.89:21.47:23.05:23.9:3.61:12.15:2.36	Anti-biofilm	[127]
*Zooglea* sp. KCCM100376	Seaweed Undaria	CBP, Glc:Gal:Man 1:2:2 WSP, Glc:Gal:Man 2:2:3	Water-holding capacity	[39]

**Table 3:** Examples of Marine Archaebacterial Exopolysaccharides.

Microorganism	Source	EPS Structure or Monosaccharide Composition	Functions and Applications	Reference
***Haloarcula***				
*H. japonica* T5 halophile	Marine saltern	Man:Gal:GlcA 2:1:3	-	[172]
*H. japonica* T6–T7 halophile	Marine saltern	Man:Gal:Glc:GlcA 1:0.2:0.2:traces	-	[172]
*H. hispanica* ATCC 33960 halophile	Solar saltern	Man:Gal:Glc 55.9:43.2:0.9 Sulphate groups	-	[173]
***Haloferax***				
*H. mediterranei* R-4 ATCC 33500 halophile	Salt ponds	Structure, Figure 4h	Pseudoplastic	[174,175]
*H. gibbonsii* ATCC 33959 halophile	Marine saltern Dead Sea	Structure, Figure 4i	-	[176]
*H. denitrificans* ATCC 35960 halophile	Saltern	Structure, Figure 4l	-	[177]
*Thermococcus litoralis* DSM5 473 e DSM 3638	Shallow marine Thermal spring	Man	Adhesion	[178]
